# Editorial: Decoding epigenetics in vascular health-from basic science to therapeutics

**DOI:** 10.3389/fcvm.2026.1793098

**Published:** 2026-03-13

**Authors:** Lian-Wang Guo

**Affiliations:** Division of Surgical Sciences, Department of Surgery, School of Medicine, University of Virginia, Charlottesville, VA, United States

**Keywords:** aneurysm, cardiac fibrosis, cell plasticity, chromatin regulation, m6A, therapeutic targeting, translational epigenetics, vascular inflammation

Cardiovascular disease is the leading cause of death globally. The pathologies arise from complex interactions among genetic susceptibility, environmental exposures, and dynamic cellular responses within the vessel wall and heart. A defining feature of cardiovascular disease is maladaptive cellular plasticity, whereby endothelial cells, vascular smooth muscle cells, immune cells, and cardiac fibroblasts adopt disease-associated phenotypes that promote inflammation, pathological remodeling, fibrosis, and structural failure. Increasing evidence indicates that these phenotypic transitions are epigenetically regulated rather than genetically fixed.

Epigenetic regulation encompasses chromatin-modifying enzymes, DNA and RNA modifications, non-coding RNAs, and metabolic influences. Rather than acting as static transcriptional regulators, these mechanisms function dynamically and in a cell type- and context-dependent manner, enabling vascular and cardiac cells to integrate inflammatory, metabolic, and biomechanical signals. Although epigenetic regulators represent attractive therapeutic targets due to the reversibility of epigenetic modifications, clinical translation has been limited by the complexity of epigenetic networks and their integration across disease states. This Research Topic, Decoding Epigenetics in Vascular Health: From Basic Science to Therapeutics, was assembled to synthesize emerging concepts and identify translational opportunities across cardiovascular disease contexts.

The four review articles included in this Topic highlight the breadth of epigenetic regulation in cardiovascular biology, spanning vascular inflammation, epitranscriptomic control, intercellular communication, and aneurysm. Together, these reviews illustrate how epigenetic mechanisms function across multiple regulatory layers and contribute to both common and disease-specific cardiovascular processes.

Inflammation is a central driver of vascular disease initiation and progression. The review “*Epigenetic modifications in vascular inflammation*” Tang et al. summarizes current evidence demonstrating how DNA methylation, histone modifications, and non-coding RNAs regulate inflammatory gene expression programs in vascular and immune cells. By linking environmental and pathological stimuli to sustained transcriptional reprogramming, this review article highlights epigenetic regulation as a key mechanism underlying persistent vascular inflammation and a potential avenue for therapeutic intervention.

Beyond chromatin-based mechanisms, RNA modifications have emerged as important regulators of cardiovascular disease. The review “*Emerging mechanisms and implications of m6A in CVDs: potential applications of natural products*” Wang et al. focuses on N6-methyladenosine (m6A), the most abundant reversible RNA modification. The authors summarize evidence for that m6A influences RNA stability, translation, and splicing, thereby modulating autophagy, apoptosis, oxidative stress, and inflammatory responses in cardiovascular disease. Of particular translational interest, this review discusses the ability of natural products to modulate m6A-related pathways, suggesting a potential strategy to target epitranscriptomic regulation with improved safety profiles.

Pathological cardiac remodeling is governed not only by cell-autonomous changes but also by disrupted communication among cardiac cell populations. In “*The epigenetic regulation of crosstalk between cardiac fibroblasts and other cardiac cell types during stress*” Kraus et al., the authors examine how epigenetic mechanisms regulate fibroblast activation and intercellular signaling with cardiomyocytes, endothelial cells, and immune cells. This review emphasizes that epigenetic control of cell–cell communication plays a critical role in determining the trajectory of cardiac remodeling and functional decline during stress and injury.

Abdominal aortic aneurysm (AAA) represents a vascular disease with high mortality yet without approved pharmacological treatment options. The review “*Epigenetic modifications in abdominal aortic aneurysms: from basic to clinical*” Liu et al. provides a comprehensive overview of epigenetic regulation in AAA, encompassing DNA methylation, histone modifications, non-coding RNAs, and RNA modifications. By identifying key epigenetically regulated genes and pathways associated with aneurysm development and progression, this work highlights epigenetic dysregulation as a central contributor to AAA pathogenesis and a potential source of therapeutic targets beyond surgical intervention.

Together, the articles in this Research Topic underscore several key principles. Epigenetic regulation in cardiovascular disease is highly context dependent, varying across cell types, disease stages, and local microenvironments. Pathogenic epigenetic programs act across multiple regulatory layers, necessitating integrative approaches that combine chromatin, RNA, metabolic, and cellular interaction networks. Despite ongoing challenges, epigenetic and epitranscriptomic regulators are promising targets for the development of precision cardiovascular therapies.

By integrating mechanistic insights with disease-focused perspectives, this Research Topic aims to support continued progress toward translating epigenetic discoveries into clinically relevant strategies for cardiovascular medicine.

